# Hamstrings load bearing in different contraction types and intensities: A shear-wave and B-mode ultrasonographic study

**DOI:** 10.1371/journal.pone.0251939

**Published:** 2021-05-19

**Authors:** Pavlos E. Evangelidis, Xiyao Shan, Shun Otsuka, Chi Yang, Takaki Yamagishi, Yasuo Kawakami

**Affiliations:** 1 Faculty of Sport Sciences, Waseda University, Tokyo, Japan; 2 Japan Society for the Promotion of Science, Tokyo, Japan; 3 First Affiliated Hospital of Sun Yat-sen University, Guangzhou, China; 4 Graduate School of Sport Sciences, Waseda University, Tokyo, Japan; Federation University Australia, AUSTRALIA

## Abstract

The main aim was to examine the load bearing of individual hamstring muscles in different contraction types and intensities, through local stiffness measurement by shear wave elastography (SWE). A secondary aim was to examine the relationship between the SWE stiffness measure and hamstrings morphology. Ten healthy males (age 22.1±4.1 years; height 173.7±5.9 cm; body mass 68.6±12.4 kg; mean ± SD) performed knee flexions on an isokinetic dynamometer at different intensities (20–70%MVC, random order) in three separate, randomized conditions: isometric (ISO), concentric (CON) and eccentric (ECC). SWE was used to measure muscle shear wave velocity (SWV) in biceps femoris long head (BFlh), semitendinosus (ST) and semimembranosus (SM) during contraction. Muscle anatomical cross-sectional area (ACSA) was measured with magnetic resonance imaging and muscle architecture with B-mode ultrasonography. Muscle SWV increased linearly with contraction intensity, but at a varying rate among muscles and contraction types. ST exhibited greater SWV than BFlh and SM in all contraction types, however, there was an upward shift in the SM SWV–torque relationship in ECC compared to ISO and CON. Strong negative correlations were found between peak ISO SWV and ST ACSA (r = -0.81, p = 0.005) and BFlh pennation angle (r = -0.75, p = 0.012). These results suggest that ST has a primary role in hamstrings load bearing in all contraction types, likely due to its morphology; however, there is evidence of increased contribution from SM in eccentric muscle actions.

## Introduction

Hamstring muscle strain injuries present a frequent and persistent impediment in sprint-related sports [1, 2], with the majority of these injuries occurring in the biceps femoris long head muscle (BFlh) [3, 4]. While it remains unclear why this muscle is more susceptible than the other biarticular hamstring muscles [i.e. semitendinosus (ST) and semimembranosus (SM)], strain injuries may occur due to excessive localized strains in BFlh [5, 6]. It is possible that excessive localized strains could result from an imbalanced load distribution and force production among the individual hamstring muscles, subjecting BFlh to loads beyond its safety limit and eventually leading to failure. However, identification of any adverse load distribution would first require an understanding of the normal muscle load distribution and how it is affected by contraction type and intensity.

As direct measurement of individual muscle forces in vivo is not feasible, some information about the load distribution pattern in hamstrings can be inferred from surface electromyography (sEMG) and magnetic resonance imaging (MRI) studies. Such studies reveal higher neural activation in medial hamstrings (i.e., ST / SM) compared to BFlh during high intensity knee flexion-dominant exercises [7–9], although the opposite pattern has also been observed [10]. Similarly, changes in MRI T2 relaxation time (i.e., proxy measure of muscle metabolic activity) suggest higher activity in ST than BFlh and SM [11, 12]. Nevertheless, sEMG signal is affected by crosstalk and movement artifacts that confound the relationship between neural activation and muscle force [13], limiting any inferences about the specific contribution of each of the medial hamstrings in load bearing. While MRI offers good spatial resolution, it is limited to post-exercise measurements, providing information only for the overall load borne by each muscle. Over the last decade, ultrasound shear wave elastography (SWE) has been increasingly used as a proxy measure of local muscle stiffness by measuring the propagation velocity of induced shear waves (SWV) within the tissue (for a detailed description see [14, 15]). Higher SWV (or shear/Young’s modulus derived from SWV) indicates a stiffer tissue [16] and it is closely related to muscle activation and force in isometric contractions [17–20]. As active muscle stiffness is directly associated with the number of attached cross-bridges [21], SWV could reflect individual muscle force changes more directly compared to sEMG and MRI measures.

To date, only one study has used SWE to describe the active muscle stiffness changes with contraction intensity in hamstrings [22], albeit only in BFlh and ST. Mendes et al. [22] found that shear modulus increased curvilinearly with torque in both BFlh and ST during submaximal isometric knee flexions. ST exhibited higher active stiffness than BFlh at low intensity (≤30%MVC), but there were no differences at higher intensities (40–60%MVC) [22]. In contrast to previous studies using EMG and MRI measures [7–9, 11, 12], these SWE data suggest that BFlh becomes increasingly important as load increases. Nevertheless, the contribution of the SM in hamstrings load bearing still needs to be clarified. SM has the largest physiological cross-sectional area among the hamstrings [23], and thus it is expected to be a major contributor in load bearing, particularly at high contraction intensities.

The relationship between active muscle stiffness and force depends on contraction type, being higher in eccentric than isometric contractions [24], suggesting that the load distribution pattern among synergists may also depend on contraction type. Although this notion is not supported by current sEMG studies [7, 8, 25], increased SM recruitment (assessed with both sEMG and T2 MRI) has been reported in eccentric compared to concentric contractions in hip-dominant exercise [26]. Nevertheless, no study has examined the effect of contraction type on active muscle stiffness using SWE. This would provide valuable information on hamstring load distribution, especially during eccentric muscle actions which are relevant to strain injuries [27].

Despite their functional synergy, hamstring constituent muscles exhibit a markedly diverse morphology [23]. As muscle size and architecture are major determinants of muscle force capacity, they are expected to directly influence hamstrings mechanical properties and load distribution. However, this has not been examined directly.

The main aim of the present study was to examine the load bearing of the individual hamstring muscles in different contraction types and intensities, using shear wave elastography (SWE). A secondary aim was to examine the relationship between SWV and hamstrings morphology (muscle size and architecture). Based on current evidence, we hypothesized that hamstrings SWV would increase with contraction intensity and reflect the activation pattern typically seen in knee flexions, i.e., greater response in ST than BF and SM. Also, we hypothesized that SWV would be greater in eccentric compared to isometric and concentric muscle actions.

## Materials and methods

### Participants

Ten young, healthy males (age 22.1±4.1 years; height 173.7±5.9 cm; body mass 68.6±12.4 kg; mean ± SD) participated in this study. All participants were recruited from Waseda University Tokorozawa campus with public announcements between April-June 2018. Exclusion criteria were any previous hamstring strains and knee joint injuries. Participants were recreationally active (iPAQ short version score 1742±1372 MET-minutes/week; https://sites.google.com/site/theipaq/home) [28]. Prior to the completion of the health screen and physical activity questionnaires, participants provided written informed consent for their participation in the study which was performed in line with the Declaration of Helsinki principles and was approved by the University Ethics Committee on Human Research.

### Overview

Participants visited the lab (Waseda University, Tokorozawa campus) on 4 separate occasions at least 7 days apart. They were instructed to avoid any strenuous physical activity for at least 2 days before each visit. In their first visit, anthropometric data were recorded, and participants were fully familiarized with the procedures of the three main sessions. In the three main sessions, participants performed a series of maximal and submaximal knee flexions on an isokinetic dynamometer while ultrasound shear wave elastography (SWE) and B-mode measurements were taken from the BFlh, ST and SM. All three main sessions were identical except for the contraction mode [isometric (ISO), concentric (CON) and eccentric (ECC)]. Finally, hamstrings anatomical muscle cross-sectional area (ACSA) was measured with magnetic resonance imaging (MRI) at the beginning of the first main session. All measurements were performed on the right leg, which was the dominant leg (defined as the preferred leg when kicking a ball) for the majority of the participants (9/10).

### Measurements

The participants lay prone on a minimally padded, custom-made wooden board placed over the isokinetic dynamometer (Con-Trex MJ, CMV AG, Dübendorf, Switzerland) that fixed the hip joint angle at 30° of flexion from full extension. This angle was selected for its relevance to the hip joint kinematics during the late swing phase in sprinting, which is considered as a high injury-risk phase of the sprint cycle [29]. To minimize any extraneous movements, two inelastic straps were placed across the pelvis, with additional straps over the torso and just above the knee joint. A strap was also placed over the extended non-involved leg. The lateral femoral condyle of the right leg was carefully aligned with the dynamometer rotational axis during an isometric knee flexion at the participants’ perceived 50% maximal voluntary torque. The dynamometer’s shin brace was placed posterior to the shank ~2 cm above the medial malleolus and the shank was tightly secured to the dynamometer lever arm. A range of motion (ROM) of 40° from full extension was set for all participants, as it corresponds to the knee joint angles during the late swing phase and it includes the knee flexors’ angle of peak torque (i.e., ~25–35° from full extension) [30, 31]. This ROM was used in the CON and ECC conditions, while the crank angle was set at 30° from full extension for the ISO condition. Following five preconditioning cycles of passive knee flexion and extension at a crank angular velocity of 8° s^-1^, the passive torque and SWV of each muscle was recorded in duplicate. In the dynamic conditions, the preset crank angular velocity was 8° s^-1^ while torque during contractions was corrected by subtracting the passive torque over the ROM. In ISO, active torque was corrected by subtracting the passive torque recorded over the preceding 1 s. In all conditions, participants completed a standardized warm-up of six submaximal contractions with progressively increasing intensity (3 x 50%, 2 x 70%, 1 x 90% of perceived maximal torque), followed by 2 sets of 3 maximal voluntary contractions (MVCs) with 1 min rest between repetitions and 3 min between sets. In each set, the knee flexor torque and SWV of each muscle was recorded. The contraction type of the warm-up and MVC contractions was based on the visit’s contraction type, i.e., in ISO, all contractions were isometric, in CON concentric and in ECC eccentric. Participants then rested for 5 min before performing three contractions at each of the following intensities: 20, 30, 40, 50, 60 and 70% MVC (identified as the highest instantaneous torque across the previously completed MVCs) with 1 min rest between contractions. This series of submaximal contractions was repeated after a 3-min rest. A monitor provided real-time visual feedback by displaying the participants’ torque response and a horizontal line at the target torque level which were instructed to match as quickly as possible and maintain throughout the contraction.

To match the contraction time among the conditions, participants were instructed to maintain the isometric contractions for 5 s. The order of the conditions, muscles and contraction intensity levels was randomized (and counter-balanced for the conditions and muscles). The assigned order for each participant was followed in all conditions.

### Shear wave elastography and B-mode ultrasound measurements

All ultrasound measurements were completed with an Aixplorer ultrasound scanner (SuperSonic Imagine, Aix-en-Provence, France) using a linear transducer (SL15-4, 4–15 Hz) and constant settings (musculoskeletal preset, smoothing 9, persistence high). A split screen on the scanner’s monitor allowed the simultaneous view and recording of both elastography and B-mode scans. The transducer was held firmly over the skin with minimal pressure, placed perpendicular to the skin and parallel to the fascicles’ direction. Optimal ultrasound probe orientation (i.e., tilt and rotation angle) was assumed when the aponeuroses and several fascicles were clearly visible. In the familiarization session, the optimal probe position for each hamstring muscle was marked with a surgical skin marker, while participants were provided with markers and asked to keep the marks between sessions. Additionally, ultrasound B-mode images of each muscle were taken and used as a reference for the replication of the probe location in the main sessions. During contraction, slight adjustment of the probe orientation was necessary to maintain a clear view of the fascicles. All measurements were taken at 50% thigh length (from greater trochanter to knee joint space), although for some participants the SM measurements were taken slightly more distally (~55% thigh length) due to difficulty in visualizing the fascicles during contraction. The region of interest (ROI) was placed over the center of the muscle belly as viewed on the scanner’s monitor, avoiding the superficial or any visible intramuscular aponeuroses. Care was taken to ensure that these structures were also avoided during contraction. The maximum ROI dimensions allowed by the scanner software were used to measure the SWV over the largest possible muscle area. Finally, the elastography recording was initiated ~3 s prior to any contraction to allow for the stabilization of the elastogram. All ultrasound measurements were conducted by the same investigator.

### MRI measurements

For all participants, hamstrings muscle anatomical cross-sectional area (ACSA) was examined on the right leg and measured using a 1.5T MRI scanner (Signa HDxt, GE) in supine position with the hip and knee joints fully extended. Oil capsules were placed on the lateral side of the thigh at 50% thigh length and proton density fast spin echo axial-plane images were acquired from the thigh with an 8-channel body array coil in a single block (imaging matrix 512x512, field of view 26 cm, spatial resolution 0.508 mm x 0.508 mm, echo time 32.4 ms, repetition time 5000 ms, slice thickness 4 mm).

### Data analysis

#### Isokinetic dynamometry data

The dynamometer torque, crank angle and velocity signals were sampled at 2000 Hz with an A/D converter (PowerLab, ADInstruments, Australia) and filtered with a 4^th^ order Butteworth-type filter with a cut-off frequency of 14 Hz for torque and 4 Hz for crank angle/velocity signals. These cut-offs were selected using residual analysis [32]. A foot pedal, connected to both the ultrasound scanner and the PowerLab, was used to initiate the recording of the SWE measurement and a pulse was generated in the PowerLab that was used to synchronize the elastography and dynamometry data.

For the dynamic contractions, the acceleration and deceleration phases were excluded and the isovelocity phase was identified as ±10% of the prescribed crank angular velocity. Only data within the isovelocity phase were used in further analyses. In all conditions, the highest instantaneous torque recorded across the six MVCs was considered as the knee flexor maximum torque for that condition.

For the submaximal contractions, torque data exceeding 90% of the target torque were averaged across the range of motion for the dynamic conditions and over the 5-s contraction for the ISO condition. All (duplicate) torque data at every intensity level were used for the regression fits while the average across the duplicate measurements was used for further statistical analysis.

#### Shear wave elastography data

The recorded SWE videos were exported and analyzed with a MATLAB custom script (MathWorks Inc, Natick, USA). The analysis involved drawing the largest possible rectangular ROI within the recorded elasticity map, avoiding any rejection areas and/or artefacts, and the SWV was calculated for every second. In the occasion of signal-saturated pixels, they were included in the ROI. This approach was selected as our pilot tests showed that signal saturation was limited and present only during ST measurements, typically at contractions ≥50%MVC. Inclusion of signal saturated pixels will result in SWV underestimation. Nevertheless, this approach did not have any significant impact in our results as the highest recorded SWV in this study was 15.7 m/s compared to an upper limit of 16.3 m/s, in a single case (participant 9; muscle ST; contraction type ECC; contraction intensity 60%MVC), while the majority of all of the recorded SWV values was below 15 m/s. Also, both our pilot data and the data presented here, exhibited a linear response as expected from the literature. Had extensive signal saturation happened, the recorded SWV would have been much closer to or would have even matched the upper measurement limit, while the SWV–torque relationship would have exhibited a clear plateau at high intensities, deviating from the linear trend. The SWV recordings obtained during the MVCs were excluded from further analyses due to excessive probe movement and extensive signal saturation. In CON and ECC, passive SWV was calculated as the average SWV recorded over the ROM while the limb was passively moved through the respective phase. In ISO, passive SWV was recorded at 30° from full extension. For the submaximal contractions, all SWV values within 10% of the peak were averaged to obtain the representative value. All duplicate SWV data at every intensity level were used for the regression fits while the average across the duplicate measurements was used for further statistical analysis.

#### Muscle architecture

Pennation angle (PA) and muscle thickness (MT) were measured in BFlh and SM muscles, while fascicle length (FL) was estimated using trigonometry (FL = MT/sin(PA)) [33]. ST muscle architecture was not measured due to the parallel orientation of the fascicles relative to the aponeuroses at the level of measurement.

In BFlh, PA was measured as the angle between the fascicles and the deep aponeurosis. In SM, PA was measured as the angle between the fascicles and the superficial aponeurosis, as they were not clearly visible near the deep aponeurosis. Two images of each muscle at rest were chosen and, within each image, three clearly defined fascicles were selected for further analysis. For each muscle, the average PA across all images and fascicles was used as the representative value. MT was measured as the perpendicular distance between the superficial and deep aponeuroses at three different sites along the imaged muscle. The average MT value across the sites and images was used in further analyses. FL was estimated in each of the two images, using the average MT in that image, and the representative FL was the average value across the two images.

#### Muscle size

BFlh, ST and SM muscle ACSA was manually outlined with Horos open-source software (v3.3.4, https://horosproject.org/). To establish the reliability of the ACSA measurement, all MR images were reanalyzed. All measurements were performed by the same investigator.

### Statistics

Descriptive data are presented as mean ± SD. The absolute reliability of the measurements was examined with the typical error between duplicate measurements expressed in measurement units (TE, mean of SD_within-subjects differences_/**√**2) and as a percentage of the respective mean (%TE, (TE/Mean_test 1–2_)x100). The intraclass correlation coefficient (ICC) was calculated (2-way mixed-effects model with absolute agreement measures) for the examination of the relative reliability of the SWV measurements.

Linear regressions were fitted to every participant’s duplicate data and to the duplicate group means. One-sample t-tests were used to examine if the regression slopes were different from zero and one-way repeated measures analysis of variance (ANOVA) to compare among muscles and contraction types.

All further statistical analyses were done on the averages of the duplicate data. Normal distribution of the data was examined with the Shapiro-Wilk test and the sphericity assumption with Mauchly’s test. When the sphericity assumption was violated, the Greenhouse-Geisser correction was applied. Three-way repeated ANOVA was used to examine the effect of contraction type, muscles and contraction intensity on SWV. Significant main effects and/or interactions were followed with two-way repeated measures ANOVAs (factors; muscles x contraction intensity) and one-way ANOVAs (factor; contraction intensity / muscles) and post hoc pairwise comparisons with Bonferroni correction whenever necessary. Bivariate correlations were examined with the Pearson product-moment correlation coefficient. The level of significance was set at p< 0.05. All statistical analyses were performed with IBM SPSS Statistics v25 (IBM Corp., Armonk, NY, USA).

## Results

### Reliability of the measurements

#### Torque (n = 10)

Torque between duplicate contractions at every contraction intensity had very high intra-session reliability across all intensity levels, muscles and contraction modes (TE≤ 2.8 Nm, %TE≤ 5.1%).

#### Shear Wave Velocity (SWV) (n = 10)

Intra-session duplicate measurements of muscle SWV exhibited moderate-to-high absolute reliability with %TE< 15% across all conditions and muscles. The relative reliability was moderate to high with ICC values primarily >0.70, albeit with wide 95%CIs (see S1–S3 Tables).

#### Muscle architecture (n = 10)

The variability of the BF and SM architecture measurements between the two analyzed images for each muscle was TE(%TE): PA, BFlh 1.0° (7%), SM 1.2° (7.3%); FL, BFlh 8.7 mm (8.2%), SM 6.0 mm (7.5%).

#### Muscle size (n = 10)

The ACSA measurement was highly reliable with TE(%TE) for BFlh 0.22 cm^2^ (1.7%), ST 0.14 cm^2^ (1.4%), and SM 0.14 cm^2^ (2.0%).

### Passive hamstrings SWV

Passive ST SWV was higher than BFlh in all contraction types (ISO, p = 0.019; CON, p< 0.001; ECC, p = 0.006, S1 Fig) but not different from SM. Similarly, SM SWV was higher than BFlh but only in CON (p = 0.004). Across contraction types, there was no difference in passive BFlh SWV, but ST and SM exhibited higher passive SWV in CON compared to ISO (ST, p = 0.004; SM, p< 0.001) and ECC (ST, p = 0.002; SM, p = 0.015).

### Active hamstrings SWV- torque relationship

**Fig 1:**
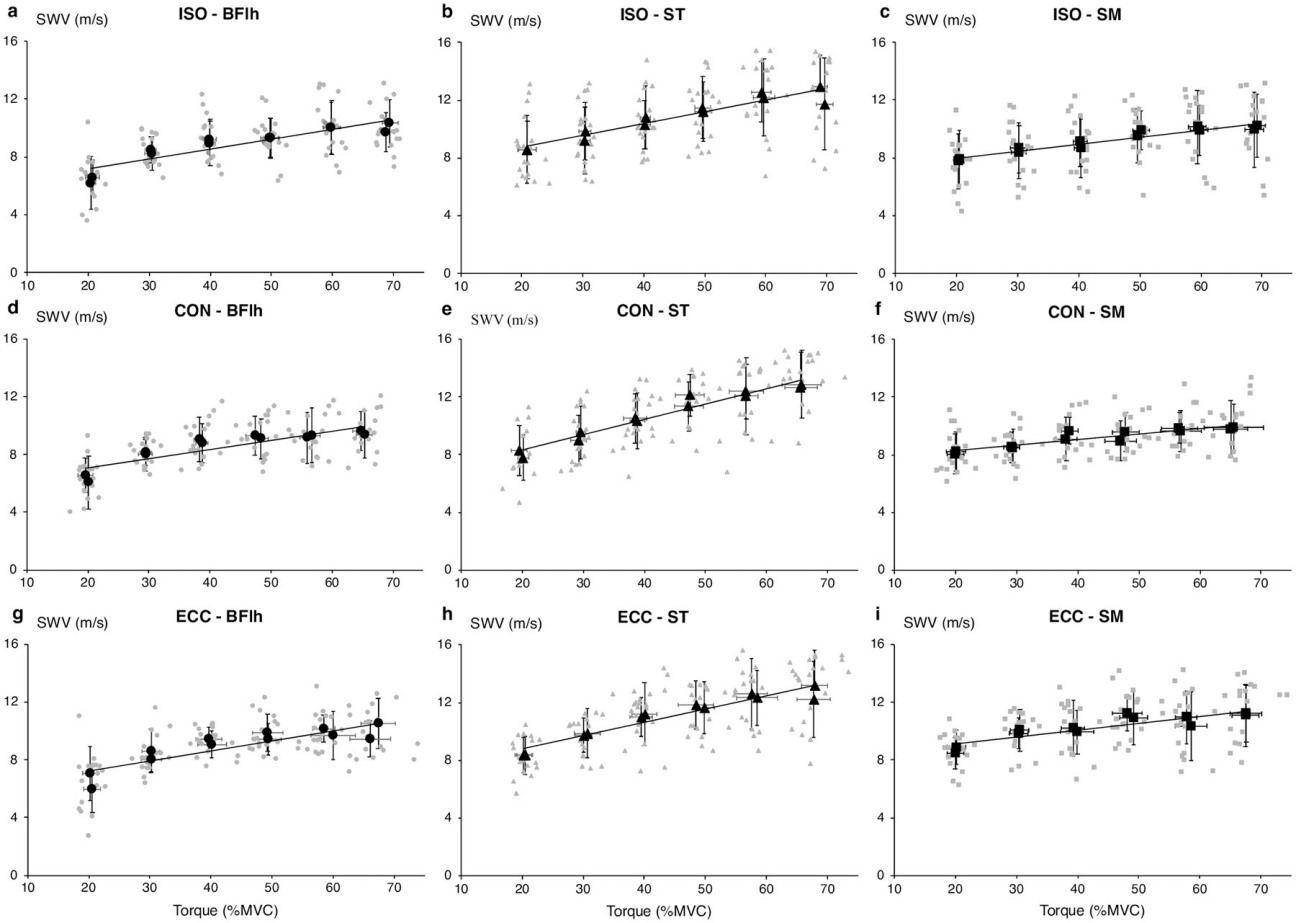
Hamstrings shear wave velocity–torque relationship. Hamstrings shear wave velocity (SWV)–torque relationship in absolute values for isometric (ISO, panels a-c), concentric (CON, panels d-f) and eccentric (ECC, panels g-i) conditions. The grey markers correspond to all duplicate contractions and the black markers correspond to the group average (SD) for each set of the duplicate contractions. The black lines correspond to the linear regressions fitted to the group averages. BFlh, biceps femoris long head; ST, semitendinosus; SM, semimembranosus.

The SWV–torque relationships for all hamstrings and contraction types derived from all (duplicate) contractions are presented in absolute and normalized to peak SWV values (Fig 1 and S2 Fig respectively). Table 1 presents the parameters of the regressions fitted to each participant’s duplicate data and to the duplicate group means.

**Table 1:** Parameters of the linear regressions fitted to each participant’s duplicate SWV–torque data and to the duplicate group means for each muscle and contraction type.

	**Individual regressions—mean (SD)**								
	ISO			CON			ECC		
	Slope	Intercept	Adj. R^2^	Slope	Intercept	Adj. R^2^	Slope	Intercept	Adj. R^2^
BFlh	0.069 (0.048)	5.8 (1.9)	0.544 (0.298)	0.063 (0.041)	5.8 (1.5)	0.514 (0.227)	0.069 (0.042)	5.9 (1.7)	0.444 (0.282)
ST	0.083^†^ (0.031)	7.1 (2.1)	0.628 (0.189)	0.105*^c^ (0.036)	6.2 (1.6)	0.728 (0.204)	0.091^c^ (0.032)	7.0 (0.9)	0.686 (0.222)
SM	0.048 (0.038)	7.0 (2.1)	0.538 (0.340)	0.038^b^ (0.028)	7.5 (1.4)	0.437 (0.324)	0.046^b^ (0.028)	8.2 (1.0)	0.431 (0.364)
	**Group regressions**								
	ISO			CON			ECC		
	Slope	Intercept	Adj. R^2^	Slope	Intercept	Adj. R^2^	Slope	Intercept	Adj. R^2^
BFlh	0.069	5.8	0.810	0.063	5.8	0.755	0.069	5.9	0.742
ST	0.083	7.1	0.945	0.105	6.2	0.946	0.092	7.0	0.917
SM	0.049	7.0	0.917	0.038	7.5	0.823	0.047	8.2	0.746

ISO, isometric; CON, concentric; ECC, eccentric; BFlh, biceps femoris long head; ST, semitendinosus; SM, semimembranosus. Adj. R^2^, adjusted coefficient of determination. Regression slopes for all muscles and contraction types are different from zero (p≤ 0.003);

* Different from ISO;

^†^ Different from CON;

^b^ different from ST;

^c^ different from SM; all p< 0.05.

**Fig 2:**
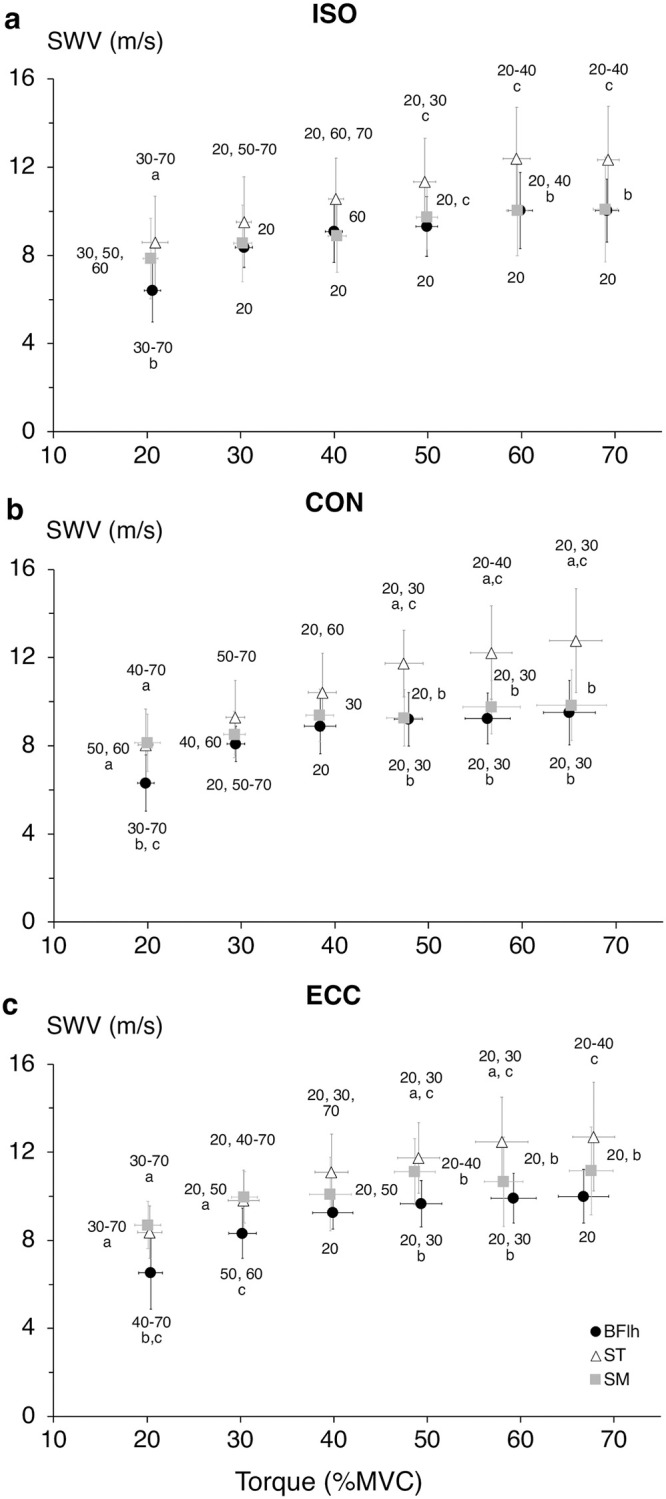
Between-muscles comparison of the hamstrings shear wave velocity–torque relationship for each contraction type. Comparison of the shear wave velocity (SWV)–torque relationship across the hamstrings in isometric (a), concentric (b) and eccentric (c) conditions. Data are presented as mean ± SD of the average across the duplicate measurements. Biceps femoris long head (BFlh, circles), semitendinosus (ST, triangles) and semimembranosus (SM, squares). Numbers denote difference from the respective intensity level in each muscle. ^a^ different from BFlh; ^b^ different from ST; ^c^ different from SM, p< 0.05.

**Fig 3:**
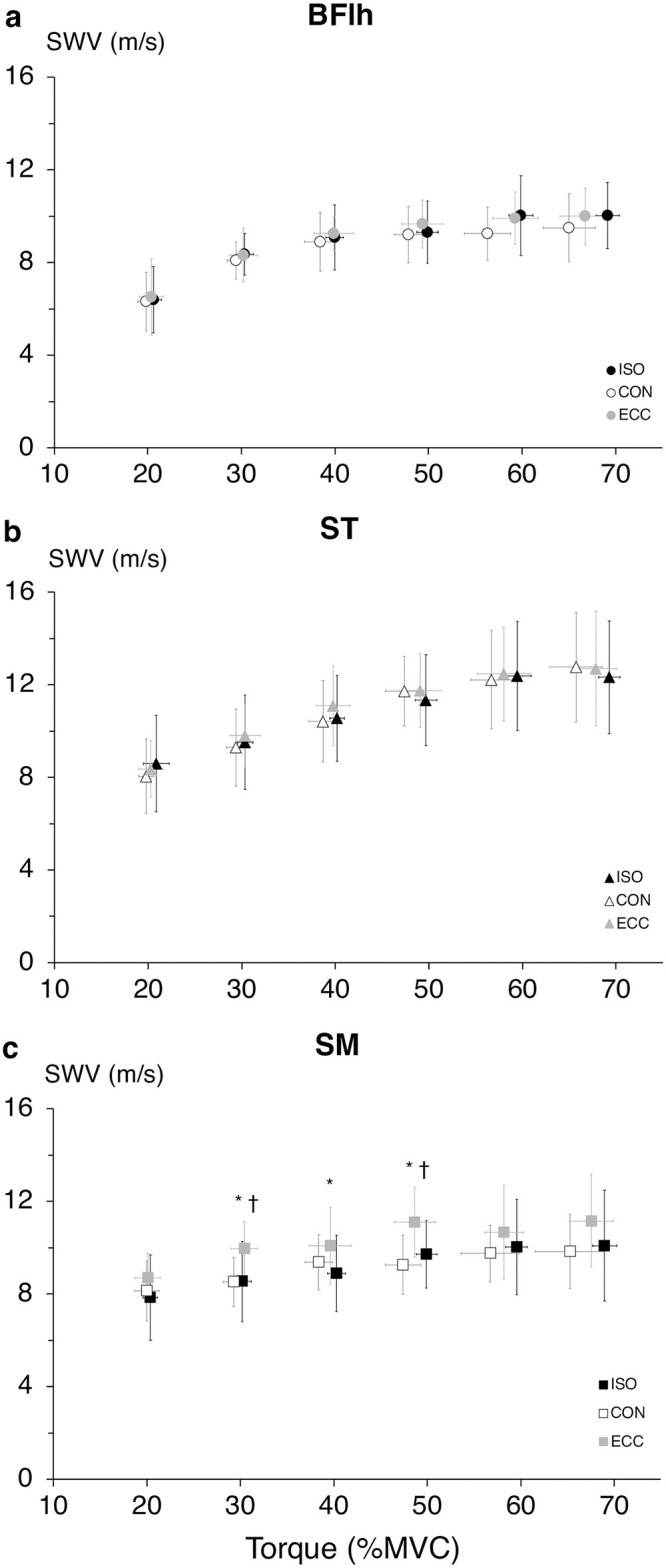
Within-contraction type comparison of the shear wave velocity–torque relationship for each hamstring muscle. Comparison of the shear wave velocity (SWV)–torque relationship in biceps femoris long head (a), semitendinosus (b) and semimembranosus (c) across the isometric (ISO, black), concentric (CON, white) and eccentric (ECC, grey) conditions. Data are presented as mean ± SD of the average across the duplicate measurements. * different from ISO, ^†^ different from CON, p< 0.05.

The overall ANOVA showed a significant muscle-by-contraction intensity interaction effect (F_10,90_ = 5.140, p< 0.001), along with significant main effects for contraction type (F_2,18_ = 3.747, p = 0.044), muscle (F_1.3,11.8_ = 12.614, p = 0.003) and intensity (F_5,45_ = 138.716, p< 0.001). Further two-way ANOVAs showed significant muscle-by-intensity interaction effects (p≤ 0.002) as well as significant main effects for muscle (p≤ 0.007) and intensity (p< 0.001) in all contraction types. Pairwise comparisons revealed that ST SWV changes were significant up to a higher contraction intensity compared to BFlh and SM, in all contraction types (Fig 2). Also, comparisons across muscles showed that ST SWV was higher compared to BFlh and SM, especially at intensities >50%MVC. Across contraction types, SM SWV was higher in ECC compared to ISO at 30, 40 and 50%MVC (p = 0.008, p = 0.022 and p = 0.015 respectively) and higher than CON at 30 and 50%MVC (both p< 0.001) (Fig 3). In contrast, there was no difference in BFlh and ST SWV among contraction types.

### Hamstrings morphology and relationship with SWV

#### Muscle size

**Fig 4:**
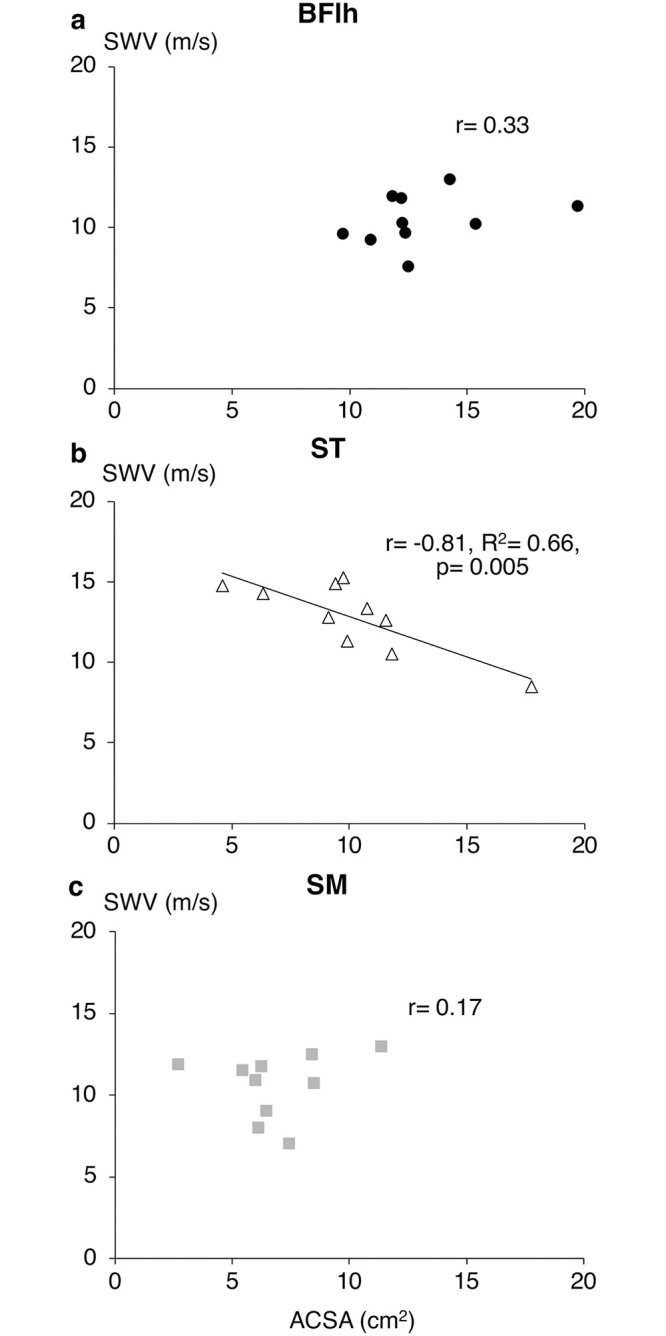
Relationships between hamstrings muscle size and shear wave velocity. Bivariate correlations between muscle anatomical cross-sectional area (ACSA) and peak isometric shear wave velocity (SWV) for biceps femoris long head (a), semitendinosus (b) and semimembranosus (c).

Muscle ACSA was 13.1 ± 2.8 cm^2^ for BFlh, 10.1 ± 3.5 cm^2^ for ST and 6.9 ± 2.3 cm^2^ for SM (different from BFlh, p = 0.002). ST ACSA exhibited moderate-to-high negative correlations with SWV in ISO across all intensities (r = -0.57 to -0.82), although statistical significance was reached only at 50%MVC (r = -0.65, p = 0.041), 60%MVC (r = -0.82, p = 0.004) and 70%MVC (r = -0.65, p = 0.04) as well as at peak SWV (r = -0.81, p = 0.005, Fig 4). In contrast, there was no significant correlation between ACSA and SWV for BFlh (r = -0.16 to 0.45) or SM (r = -0.29 to 0.31) at any contraction intensity.

In CON, no significant correlations were found between ACSA and SWV for any of the hamstring muscles (BFlh, r = -0.57 to -0.05; ST, r = -0.62 to -0.29; SM, r = -0.03 to 0.62). In ECC, BFlh ACSA was related to SWV only at 40%MVC (r = -0.76, p = 0.011) and ST ACSA with passive SWV (r = 0.70, p = 0.024) and SWV at 50%MVC (r = -0.66, p = 0.039). Finally, SM SWV was not related to muscle size at any intensity (r = -0.23 to 0.07).

#### Muscle architecture

**Fig 5:**
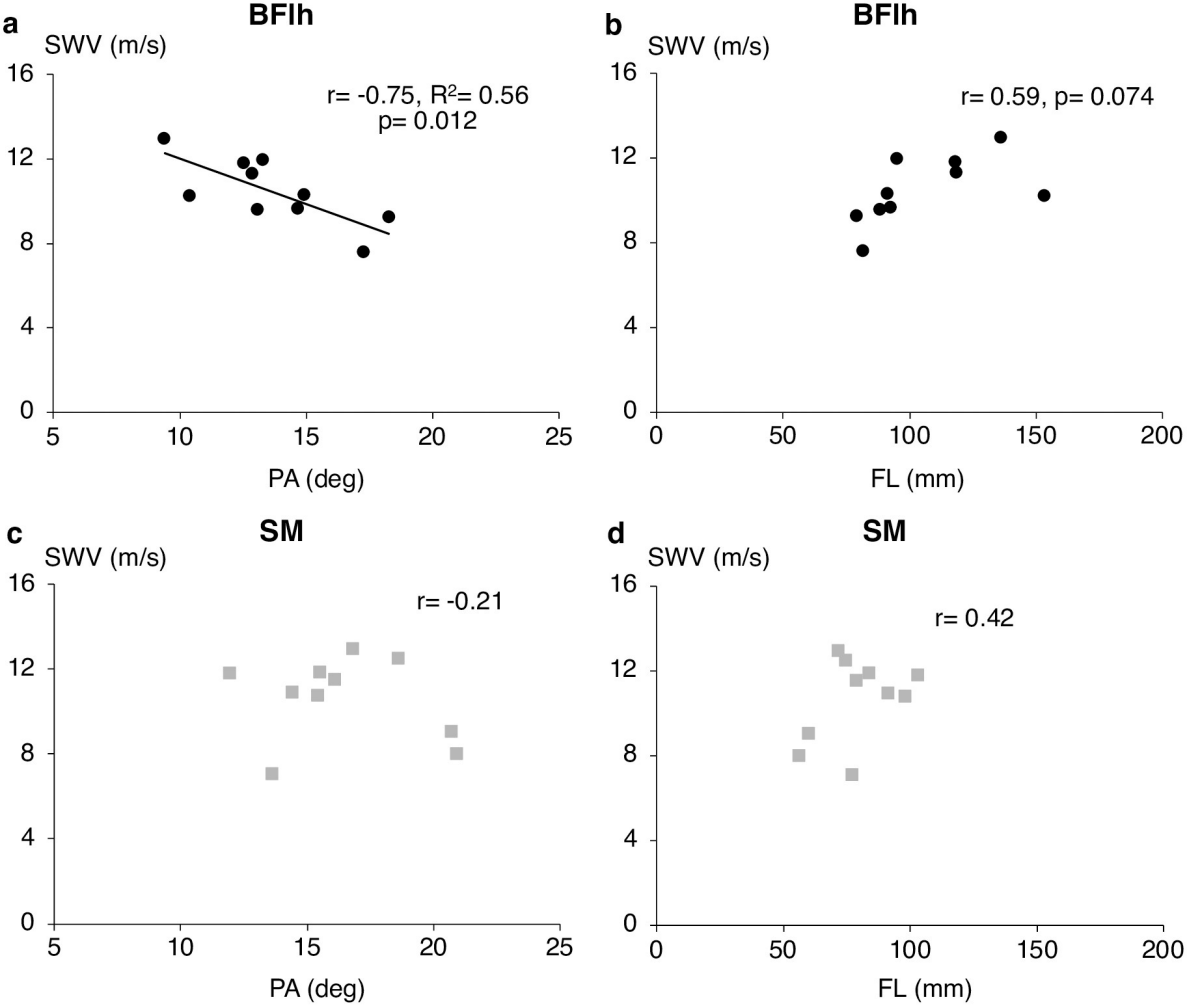
Relationships between hamstrings muscle architecture and shear wave velocity. Bivariate correlations between peak isometric shear wave velocity (SWV) and pennation angle (PA) and fascicle length (FL) for biceps femoris long head (a, b) and semimembranosus (c, d).

BFlh exhibited higher PA (13.7 ± 2.8° vs. 16.4 ± 2.9°, p = 0.09) and longer FL (105.5 ± 21.7 mm vs. 79.8 ± 15.2 mm, p = 0.021) than SM. In ISO, BFlh PA was strongly correlated with SWV at 50%MVC (r = -0.71, p = 0.022), 60%MVC (r = -0.71, p = 0.021), 70%MVC (r = -0.75, p = 0.013) and peak SWV (r = -0.75, p = 0.012, Fig 5), but there were no significant relationships between SM PA and SWV at any intensity (r = -0.49 to -0.05). BFlh and SM PA was not related to their respective SWV in either CON or ECC (BFlh, CON, r = -0.11 to 0.21, ECC, r = -0.44 to 0.28; SM, CON, r = -0.27 to -0.05, ECC, r = -0.54 to 0.46).

For BFlh, FL was strongly related to ISO SWV only at 70%MVC (r = 0.72, p = 0.02) but there was no relationship with CON or ECC SWV at any intensity (CON, r = -0.40 to -0.06; ECC, r = -0.44 to 0.29). No correlation was found between SM FL and SWV in any condition and contraction intensity (ISO, r = 0.20 to 0.52; CON, r = -0.01 to 0.26; ECC, r = -0.20 to 0.50).

## Discussion

The aim of this study was to examine the load bearing of the individual hamstring muscles in different contraction types and intensities, through local stiffness measurement as SWV by using shear wave elastography. A secondary aim was to examine the relationship between SWV and hamstrings morphology. We found that SWV increased linearly with contraction intensity, exhibiting a varying rate across muscles and contraction types. ST SWV was higher than BFlh and SM especially at high intensities, and this pattern remained largely consistent across contraction types. Nevertheless, SM SWV–torque relationship exhibited an upward shift in ECC compared to ISO and CON. Finally, ST muscle size and BFlh PA were negatively correlated with their respective peak isometric SWV, but hamstrings muscle morphology was unrelated to active stiffness in dynamic contractions.

Our results suggest that, during knee flexion, hamstrings load is primarily borne by ST followed by BFlh and SM, and this order is largely maintained in all contraction types. This pattern of load distribution (ST > BFlh and SM) is in line with evidence based on EMG and functional MRI data [7–9, 11, 12]. Such a pattern suggests that ST, which has the smallest PCSA and torque-generating capacity among the biarticular hamstrings [23, 34], sustains a relatively greater load than the larger and stronger BFlh and SM. While this is potentially a less efficient strategy [35], it may reflect hamstrings’ highly diverse architecture. The fusiform ST can transmit forces more efficiently along the line of muscle action, while its long fascicles imply that their sarcomeres operate closer to their optimal length over a larger ROM [36]. As a result, the number of attached cross-bridges per activation level is (near-)maximal leading to higher active stiffness compared to the pennate BFlh and SM. The central role of ST in hamstrings load bearing during knee flexion is further corroborated by its higher rate of SWV increase (i.e., slope) across all contraction types, especially compared to SM. On the other hand, BFlh and SM lower SWV may reflect their larger PCSA and force-generating capacity that would require a lower level of neural activation to achieve a certain level of force. It is noteworthy that SM SWV was close to its peak value (>70%peak SWV) throughout the range of contraction intensities, suggesting limited changes in its load bearing contribution even at high loads.

### Effect of contraction type and intensity on SWV

This is the first study that examined the effect of contraction type on muscle SWV, and any comparison of our results is difficult. However, the SWV–torque relationship across the hamstring muscles remained largely consistent across contraction types. On a group level, all muscles exhibited linear relationships, in line with previous SWE studies in isometric contractions in a range of different muscles (first dorsal interosseous [18], biceps brachii [37, 38], abductor digiti minimi [17, 18, 39], vastus lateralis & rectus femoris [40]). In hamstrings, the only other SWE study [22] reported a plateau in isometric shear modulus after 30–40%MVC for both BFlh and ST. It is unclear why this discrepancy exists, as both studies examined comparable cohorts with similar methodologies. However, we used a flexed hip joint position (30° vs. 0°) that allowed hamstrings to operate close to their optimal length and exert higher forces [41, 42]. On an individual level, there was large variability in the SWV–torque relationship that was mainly due to small SWV changes with contraction intensity in some participants, rather than a deviation from a linear trend. Interestingly, individuals with minimal SWV changes in one muscle exhibited large changes in another. Nevertheless, it is noteworthy that in 7 out of 10 individuals ST SWV was the highest of all hamstrings, either at every intensity level or at the high intensities. Another interesting point is that the load bearing pattern within the same individual was relatively consistent across contraction types.

While the shape of the active muscle SWV–torque relationship was very similar across contraction types, SM SWV was higher at the 30–50%MVC range in ECC compared to ISO and CON, along with an increased—albeit not statistically significant- intercept (ECC 8.2±1.0 m/s vs. ISO, 7.0±2.1 m/s and CON 7.5±1.4 m/s), suggesting an upward shift of the SWV–torque relationship and an increased SM contribution to load bearing. This notion is consistent with the higher muscle forces and increased stiffness expected in eccentric contractions, due to the increased number of attached cross-bridges, stretching of the parallel elastic component and, theoretically, the calcium-dependent engagement of titin [43]. Contraction type also influenced the slope of the ST SWV–torque relationship, which was 26.5% higher in CON than ISO (p = 0.039) and 15.4% higher than ECC (ns). These differences indicate an increased load borne by ST during CON, consistent with the higher neural activation and greater metabolic activity typically seen in concentric compared to the other contraction types [44, 45].

Some curvilinearity is also present in our data, especially for BFlh. This appears to originate from the relatively low SWV at 20%MVC, likely due to non-uniform neural activation and low local stress at very low intensities. In addition, as SWV is estimated at a single plane, some activated muscle fascicles may have not been detected, further contributing to the low SWV seen at 20%MVC. Relatively low SWV can also be seen at high intensity contractions in some participants, however, measurements at high intensities are more susceptible to artifacts and probe misalignment due to participant movement, muscle bulging and increase in pennation angle, all of which would tend to decrease the estimated SWV. Finally, it should be noted that although BFlh, ST and SM are major knee flexors, other muscles also contribute to knee flexion torque (i.e., BF short head, sartorius, gracilis, gastrocnemius), which would dissociate hamstrings SWV changes from knee flexor torque changes. This also partly explains the lower R^2^ values of the regressions in this study compared to the ones reported for small muscles acting as the only (or predominant) agonists over a joint (R^2^≥ 0.96 [17, 18, 39]).

It is possible that the differences in SWV across the hamstrings also reflect methodological limitations. The ROI dimensions were maximized to ensure a more reliable and representative measurement [17]. However, using the same absolute ROI dimensions in all muscles meant that a larger portion of the activated muscle was inadvertently captured in the small ST compared to the larger BFlh and SM, providing a more representative measurement of the former muscle. Another limitation is that SWV was not normalized to its MVC values due to excessive artifacts (see Methods). However, normalization to peak SWV across the examined intensities did not alter the SWV–torque relationship or its pattern among hamstrings. Finally, a limitation of this study is the relatively small sample size of University students examined. A larger, more variable athletic population may have been more representative of the hamstrings active SWV across contraction types and intensities. Nevertheless, our results are in general agreement with previous findings, exhibiting similar group means and variability estimates in healthy individuals [22].

### Effect of muscle morphology on SWV

Muscle morphology exhibited a muscle- and contraction type-specific relationship with SWV. The strong negative correlation between ST ACSA and peak ISO ST SWV, along with no significant correlations for BFlh and SM, supports the notion that ST is a primary load bearer among the hamstrings, uncoupling the load distribution pattern from the force-generating capacity of the muscles. If this is the case, then individuals with small ST would have their muscles operating closer to their absolute load bearing capacity, and therefore more susceptible to fatigue and overload injuries. Interestingly, this notion could also explain the efficacy of the Nordic hamstring exercise in hamstrings strain injury prevention [46], as it causes a greater hypertrophic response in ST compared to BFlh and SM [47]. Although the vast majority of hamstring strain injuries are sustained by BFlh, this may be the result of an overloaded ST that disturbs the load distribution among hamstrings, creating large inter-muscular shearing forces. Despite being overloaded, the long, parallel ST fascicles would better protect the muscle from a strain injury, whilst the shorter BFlh fascicles would be at a more disadvantageous position to resist excessive loading and the resultant strains. Another possibility for the observed negative correlation is that it reflects a more representative SWV measurement for individuals with smaller muscles (discussed above).

BFlh PA exhibited a strong negative correlation with peak ISO SWV. There is no previous data in hamstrings but, within tibialis anterior, shear modulus estimated with SWE was not related with PA in evoked isometric contractions (r = -0.06) [48]. It is unclear why this discrepancy exists; however, a possible explanation is that the greater anisotropy in pennate muscles attenuates the shear waves propagation [49]. Nevertheless, any such negative effect is expected to be of negligible magnitude, even at pennation angles much higher than the ones measured in this study [50, 51]. The lack of any significant correlations between muscle morphology and SWV in dynamic contractions may be due to the averaging of the SWV measurement over the examined ROM, that may have blunted any length-specific changes. However, the relatively narrow ROM in this study, around the angle of peak force and strain for all hamstring muscles [42], suggests that any such changes would have a small effect.

## Conclusions

In conclusion, SWV increases linearly with contraction intensity in all biarticular hamstrings, but at a differing rate among muscles and contraction types, reflecting hamstrings’ diverse anatomy. Our results suggest that ST is a primary load bearer among the constituent hamstring muscles, irrespective of contraction type. Nevertheless, there is evidence that SM load bearing is increased in eccentric contractions. The practical implication of these findings is that hamstrings load distribution appears to be driven by ST and its muscle size may be a key factor in hamstrings strain injury prevention.

## Supporting information

S1 Fig Hamstrings passive shear wave velocity. Passive shear wave velocity (SWV) for the biceps femoris long head (BFlh), semitendinosus (ST) and semimembranosus (SM) muscles in isometric (ISO), concentric (CON) and eccentric (ECC) conditions. ^a^ different from BFlh, * different from ISO, ^#^ different from ECC, p< 0.05.
(PDF)

S2 Fig Normalized hamstrings shear wave velocity—Torque relationship. Hamstrings muscle shear wave velocity (SWV)–torque relationship normalized to peak for isometric (ISO, panels a-c), concentric (CON, panels d-f) and eccentric (ECC, panels g-i) conditions. The grey markers correspond to all duplicate contractions and the black markers correspond to the group average (SD) for each set of the duplicate contractions. The black lines correspond to the linear regressions fitted to the group averages. BFlh, biceps femoris long head; ST, semitendinosus; SM, semimembranosus.
(PDF)

S1 File Data. (XLSX)

S1 Table Descriptive and reliability statistics for hamstrings SWV in isometric condition. Descriptive statistics and absolute (TE, %TE) and relative (ICC) reliability measures for muscle shear wave velocity in isometric condition (ISO). TE, typical error; ICC, intraclass correlation coefficient; CI, confidence interval; BFlh, biceps femoris long head; ST, semitendinosus; SM, semimembranosus.
(PDF)

S2 Table Descriptive and reliability statistics for hamstrings SWV in concentric condition. Descriptive statistics and absolute (TE, %TE) and relative (ICC) reliability measures for muscle shear wave velocity in isometric condition (CON). TE, typical error; ICC, intraclass correlation coefficient; CI, confidence interval; BFlh, biceps femoris long head; ST, semitendinosus; SM, semimembranosus.
(PDF)

S3 Table Descriptive and reliability statistics for hamstrings SWV in eccentric condition. Descriptive statistics and absolute (TE, %TE) and relative (ICC) reliability measures for muscle shear wave velocity in isometric condition (ECC). TE, typical error; ICC, intraclass correlation coefficient; CI, confidence interval; BFlh, biceps femoris long head; ST, semitendinosus; SM, semimembranosus.
(PDF)
