# Supplementary figures and images for: Hamstrings load bearing in different contraction types and intensities: A shear-wave and B-mode ultrasonographic study
Source: PLoS One. 2021 May 19;16(5):e0251939. doi: 10.1371/journal.pone.0251939 (PMC8133428; doi:10.1371/journal.pone.0251939)

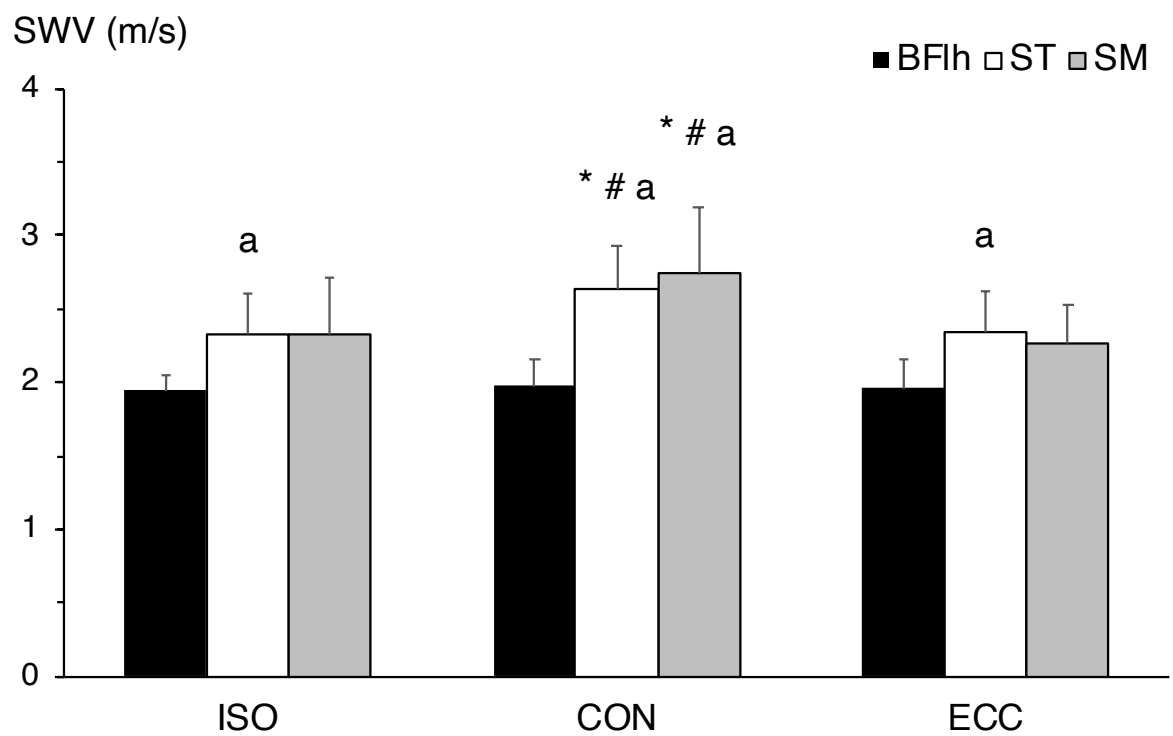

Supplement: S1 Fig — Passive shear wave velocity (SWV) for the biceps femoris long head (BFlh), semitendinosus (ST) and semimembranosus (SM) muscles in isometric (ISO), concentric (CON) and eccentric (ECC) conditions. a different from BFlh, * different from ISO, # different from ECC, p< 0.05. (PDF) [file pone.0251939.s001.pdf]

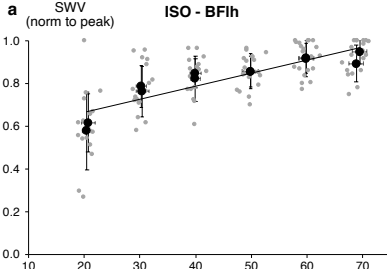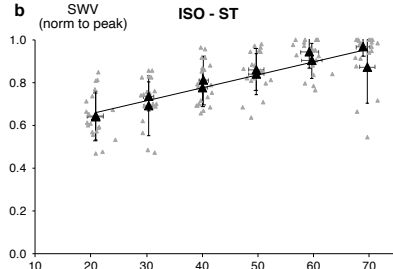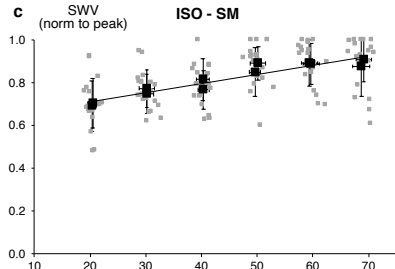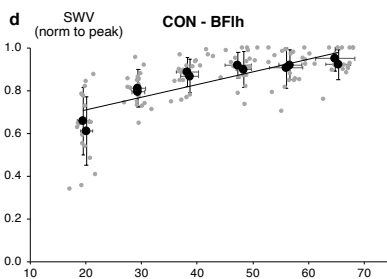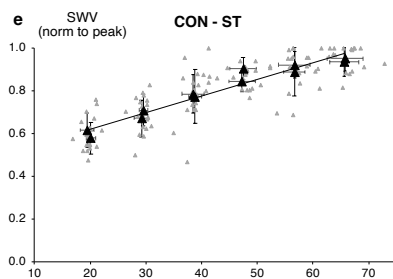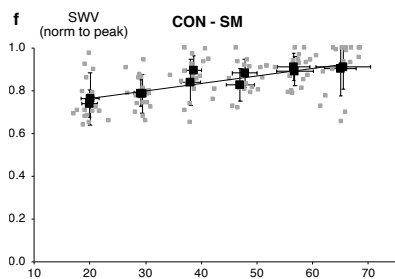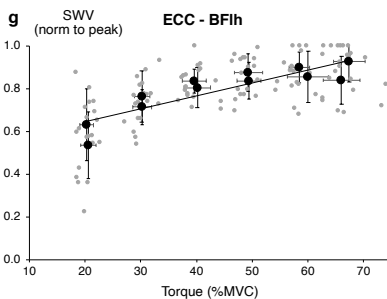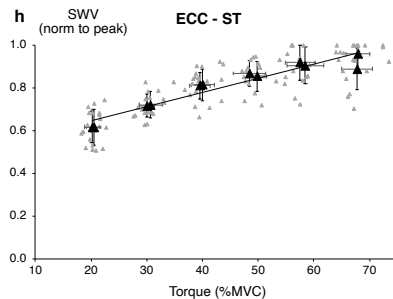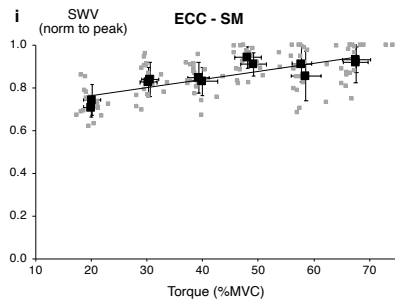

Supplement: S2 Fig — Hamstrings muscle shear wave velocity (SWV)–torque relationship normalized to peak for isometric (ISO, panels a-c), concentric (CON, panels d-f) and eccentric (ECC, panels g-i) conditions. The grey markers correspond to all duplicate contractions and the black markers correspond to the group average (SD) for each set of the duplicate contractions. The black lines correspond to the linear regressions fitted to the group averages. BFlh, biceps femoris long head; ST, semitendinosus; SM, semimembranosus. (PDF) [file pone.0251939.s002.pdf]
