# Supplementary material for: Hamstrings load bearing in different contraction types and intensities: A shear-wave and B-mode ultrasonographic study
Source: PLoS One. 2021 May 19;16(5):e0251939. doi: 10.1371/journal.pone.0251939 (PMC8133428; doi:10.1371/journal.pone.0251939)
Supplement: S1 Table — Descriptive statistics and absolute (TE, %TE) and relative (ICC) reliability measures for muscle shear wave velocity in isometric condition (ISO). TE, typical error; ICC, intraclass correlation coefficient; CI, confidence interval; BFlh, biceps femoris long head; ST, semitendinosus; SM, semimembranosus. (PDF) [file pone.0251939.s004.pdf]

	ISO Shear wave velocity (m/s)							
	Mean (SD)				Reliability measures			
	%MVC	Trial 1	Trial 2	Average	*P* value	TE (m/s)	TE (%)	ICC (95% CI)
**BFlh**	0	1.9 (0.1)	1.9 (0.1)	1.9 (0.1)	0.51	0.1	2.7	0.75 (0.27–0.93)
	20	6.6 (1.2)	6.2 (1.8)	6.4 (1.4)	0.33	0.8	13.3	0.70 (0.21–0.92)
	30	8.5 (0.9)	8.2 (1.1)	8.4 (0.9)	0.42	0.7	8.9	0.50 (-0.13–0.85)
	40	8.9 (1.5)	9.2 (1.4)	9.1 (1.4)	0.27	0.6	6.4	0.83 (0.49–0.95)
	50	9.3 (1.4)	9.3 (1.4)	9.3 (1.4)	0.90	0.3	3.5	0.95 (0.80–0.99)
	60	10.0 (1.8)	10.0 (1.9)	10.0 (1.7)	0.98	0.9	8.5	0.80 (0.38–0.95)
	70	10.4 (1.6)	9.7 (1.4)	10.0 (1.4)	0.05	0.6	6.4	0.77 (0.24–0.94)
**ST**	0	2.3 (0.3)	2.3 (0.3)	2.3 (0.3)	0.68	0.1	2.6	0.97 (0.88–0.99)
	20	8.6 (2.0)	8.6 (2.3)	8.6 (2.1)	0.94	0.8	9.5	0.87 (0.56–0.97)
	30	9.2 (2.3)	9.8 (2.0)	9.5 (2.0)	0.18	1.0	10.5	0.77 (0.34–0.94)
	40	10.3 (1.7)	10.8 (2.2)	10.6 (1.9)	0.24	0.8	8.0	0.80 (0.41–0.95)
	50	11.2 (2.1)	11.5 (2.1)	11.3 (2.0)	0.52	1.0	9.1	0.77 (0.32–0.94)
	60	12.6 (2.1)	12.2 (2.7)	12.4 (2.3)	0.36	0.8	6.5	0.89 (0.64–0.97)
	70	11.7 (3.2)	12.9 (2.2)	12.3 (2.4)	0.15	1.7	13.8	0.58 (0.03–0.87)
**SM**	0	2.3 (0.4)	2.3 (0.4)	2.3 (0.4)	0.47	0.1	3.0	0.95 (0.81–0.99)
	20	7.8 (1.9)	7.9 (1.9)	7.9 (1.8)	0.69	0.8	10.4	0.84 (0.47–0.96)
	30	8.4 (1.8)	8.7 (1.8)	8.5 (1.7)	0.25	0.6	6.5	0.89 (0.65–0.97)
	40	9.1 (1.7)	8.7 (2.0)	8.9 (1.6)	0.48	1.3	14.7	0.53 (-0.11–0.86)
	50	9.5 (1.9)	9.9 (1.4)	9.7 (1.5)	0.42	1.1	10.9	0.60 (0.01–0.88)
	60	9.9 (1.7)	10.1 (2.5)	10.0 (2.1)	0.64	0.9	8.7	0.85 (0.50–0.96)
	70	10.2 (2.2)	9.9 (2.6)	10.1 (2.4)	0.38	0.6	6.3	0.93 (0.75–0.98)
