# Supplementary material for: Hamstrings load bearing in different contraction types and intensities: A shear-wave and B-mode ultrasonographic study
Source: PLoS One. 2021 May 19;16(5):e0251939. doi: 10.1371/journal.pone.0251939 (PMC8133428; doi:10.1371/journal.pone.0251939)
Supplement: S2 Table — Descriptive statistics and absolute (TE, %TE) and relative (ICC) reliability measures for muscle shear wave velocity in isometric condition (CON). TE, typical error; ICC, intraclass correlation coefficient; CI, confidence interval; BFlh, biceps femoris long head; ST, semitendinosus; SM, semimembranosus. (PDF) [file pone.0251939.s005.pdf]

	CON Shear wave velocity (m/s)							
		Mean (SD)			Reliability measures			
	%MVC	Trial 1	Trial 2	Average	*P* value	TE (m/s)	TE (%)	ICC (95% CI)
**BFlh**	0	2.0 (0.2)	2.0 (0.2)	2.0 (0.2)	1.00	0.0	0.9	0.97 (0.89–0.99)
	20	6.6 (1.4)	6.1 (1.3)	6.3 (1.3)	0.04	0.5	7.4	0.83 (0.36–0.96)
	30	8.1 (0.8)	8.0 (1.0)	8.1 (0.8)	0.65	0.6	7.8	0.55 (-0.10–0.87)
	40	9.0 (1.5)	8.8 (1.2)	8.9 (1.3)	0.53	0.7	7.9	0.75 (0.27–0.93)
	50	9.1 (1.4)	9.3 (1.2)	9.2 (1.2)	0.53	0.7	8.1	0.70 (0.16–0.91)
	60	9.2 (1.3)	9.3 (1.4)	9.2 (1.2)	0.65	0.9	9.9	0.55 (-0.11–0.87)
	70	9.4 (1.6)	9.6 (1.4)	9.5 (1.5)	0.36	0.5	5.6	0.87 (0.57–0.96)
**ST**	0	2.6 (0.3)	2.6 (0.3)	2.6 (0.3)	0.44	0.1	1.9	0.97 (0.88–0.99)
	20	8.3 (1.7)	7.8 (1.6)	8.0 (1.6)	0.02	0.4	5.1	0.91 (0.45–0.98)
	30	9.0 (1.7)	9.6 (1.8)	9.3 (1.7)	0.15	0.8	8.6	0.78 (0.35–0.94)
	40	10.5 (1.7)	10.3 (1.9)	10.4 (1.8)	0.49	0.6	5.5	0.91 (0.69–0.98)
	50	12.1 (1.4)	11.4 (1.7)	11.7 (1.5)	0.04	0.7	6.0	0.73 (0.17–0.93)
	60	12.4 (1.9)	12.1 (2.7)	12.2 (2.1)	0.55	1.3	10.5	0.71 (0.19–0.92)
	70	12.9 (2.4)	12.7 (2.4)	12.8 (2.4)	0.46	0.5	4.1	0.95 (0.84–0.99)
**SM**	0	2.7 (0.5)	2.7 (0.4)	2.7 (0.5)	0.52	0.1	3.1	0.96 (0.84–0.99)
	20	8.1 (1.4)	8.2 (1.4)	8.1 (1.3)	0.59	0.6	7.8	0.80 (0.37–0.95)
	30	8.5 (1.1)	8.5 (1.2)	8.5 (1.1)	0.92	0.7	7.9	0.69 (0.11–0.91)
	40	9.1 (1.5)	9.7 (0.9)	9.4 (1.2)	0.07	0.6	6.7	0.69 (0.17–0.91)
	50	9.0 (1.4)	9.6 (1.3)	9.3 (1.3)	0.08	0.7	7.1	0.70 (0.19–0.92)
	60	9.7 (1.4)	9.9 (1.1)	9.8 (1.2)	0.32	0.5	4.7	0.86 (0.57–0.96)
	70	9.9 (1.6)	9.8 (2.0)	9.8 (1.6)	0.88	1.1	11.6	0.62 (-0.01–0.89)
