# Supplementary material for: Hamstrings load bearing in different contraction types and intensities: A shear-wave and B-mode ultrasonographic study
Source: PLoS One. 2021 May 19;16(5):e0251939. doi: 10.1371/journal.pone.0251939 (PMC8133428; doi:10.1371/journal.pone.0251939)
Supplement: S3 Table — Descriptive statistics and absolute (TE, %TE) and relative (ICC) reliability measures for muscle shear wave velocity in isometric condition (ECC). TE, typical error; ICC, intraclass correlation coefficient; CI, confidence interval; BFlh, biceps femoris long head; ST, semitendinosus; SM, semimembranosus. (PDF) [file pone.0251939.s006.pdf]

	ECC Shear wave velocity (m/s)							
	Mean (SD)				Reliability measures			
	%MVC	Trial 1	Trial 2	Average	*P* value	TE (m/s)	TE (%)	ICC (95% CI)
**BFlh**	0	1.9 (0.2)	2.0 (0.2)	2.0 (0.2)	0.44	0.0	2.5	0.92 (0.72–0.98)
	20	6.0 (1.6)	7.1 (1.9)	6.5 (1.6)	0.02	0.8	12.4	0.67 (0.01–0.91)
	30	8.0 (0.9)	8.6 (1.5)	8.3 (1.1)	0.08	0.6	7.7	0.67 (0.14–0.91)
	40	9.1 (0.9)	9.4 (0.8)	9.2 (0.7)	0.25	0.7	7.4	0.40 (-0.21–0.80)
	50	9.9 (1.3)	9.4 (1.1)	9.7 (1.0)	0.25	0.8	8.5	0.51 (-0.08–0.85)
	60	9.7 (1.7)	10.2 (0.9)	9.9 (1.1)	0.36	1.1	11.0	0.34 (-0.31–0.78)
	70	9.5 (1.2)	10.5 (1.7)	10.0 (1.2)	0.09	1.3	12.8	0.25 (-0.26–0.71)
**ST**	0	2.3 (0.3)	2.3 (0.3)	2.3 (0.3)	0.76	0.1	2.5	0.95 (0.81–0.99)
	20	8.4 (1.3)	8.4 (1.2)	8.4 (1.2)	0.94	0.6	7.1	0.80 (0.37–0.95)
	30	9.9 (1.7)	9.7 (1.2)	9.8 (1.4)	0.71	0.7	7.0	0.80 (0.36–0.95)
	40	11.2 (2.2)	11.0 (1.3)	11.1 (1.7)	0.62	0.8	7.5	0.80 (0.39–0.95)
	50	11.7 (1.8)	11.8 (1.7)	11.7 (1.6)	0.67	1.0	8.3	0.70 (0.17–0.92)
	60	12.3 (1.9)	12.6 (2.4)	12.5 (2.0)	0.59	1.2	9.4	0.72 (0.21–0.92)
	70	12.2 (2.6)	13.2 (2.5)	12.7 (2.5)	0.03	0.8	6.5	0.85 (0.38–0.96)
**SM**	0	2.3 (0.3)	2.3 (0.3)	2.3 (0.3)	0.59	0.1	4.2	0.92 (0.73–0.98)
	20	8.5 (1.1)	8.9 (1.2)	8.7 (1.1)	0.22	0.7	7.8	0.65 (0.13–0.90)
	30	10.1 (1.5)	9.8 (1.1)	10.0 (1.2)	0.53	0.8	7.8	0.64 (0.07–0.90)
	40	10.0 (1.5)	10.2 (1.9)	10.1 (1.7)	0.37	0.7	6.8	0.86 (0.54–0.96)
	50	11.0 (1.9)	11.3 (1.3)	11.1 (1.5)	0.40	0.7	6.7	0.79 (0.37–0.94)
	60	10.4 (2.4)	11.0 (1.9)	10.7 (2.0)	0.19	1.0	9.0	0.78 (0.37–0.94)
	70	11.1 (2.1)	11.2 (2.0)	11.2 (2.0)	0.69	0.7	6.3	0.89 (0.63–0.97)
